# Miller Fisher Syndrome With Guillain-Barré Syndrome Overlap in a 20-Month-Old Girl: A Case Report

**DOI:** 10.7759/cureus.111738

**Published:** 2026-06-29

**Authors:** Hind Zahiri, Aziza Elouali, Abdeladim Babakhouya, Maria Rkain

**Affiliations:** 1 Pediatric Neurology, Centre Hospitalier Universitaire (CHU) Mohammed VI University Hospital, Oujda, MAR; 2 Pediatric Neurology, Faculty of Medicine and Pharmacy, Mohamed I University, Oujda, MAR; 3 Pediatrics, Faculty of Medicine and Pharmacy, Mohamed I University, Oujda, MAR; 4 Pediatrics, Centre Hospitalier Universitaire (CHU) Mohammed VI University Hospital, Oujda, MAR; 5 Pediatric Gastroenterology, Centre Hospitalier Universitaire (CHU) Mohammed VI University Hospital, Oujda, MAR

**Keywords:** anti-gq1b antibodies, anti-gt1a antibodies, chronic campylobacter jejuni infection, guillain-barré syndrome, miller fisher syndrome (mfs), neurology and pediatric neurology, polyradiculoneuropathy

## Abstract

Miller Fisher syndrome (MFS) is an uncommon variant of Guillain-Barré syndrome (GBS) classically characterized by ophthalmoplegia, ataxia, and areflexia. Overlap forms with GBS are rare in early childhood and may be difficult to recognize, particularly in toddlers in whom neurological examination is limited. We report the case of a 20-month-old girl who presented with a seven-day history of progressive gait disturbance, repeated falls, refusal to walk, and convergent strabismus. Neurological examination showed severe axial ataxia, inability to sit or stand without support, generalized areflexia, symmetrical limb weakness, and right abducens nerve palsy. Brain and spinal magnetic resonance imaging (MRI) were normal. Cerebrospinal fluid (CSF) analysis showed mild albuminocytologic dissociation. Electroneuromyography demonstrated an acute motor-predominant polyradiculoneuropathy with preserved sensory responses. Anti-ganglioside antibody testing was positive for anti-ganglioside GQ1b (anti-GQ1b) and anti-ganglioside GT1a (anti-GT1a) immunoglobulin G (IgG) antibodies, supporting a diagnosis within the anti-GQ1b antibody syndrome spectrum. *Campylobacter jejuni* serology was positive despite the absence of preceding gastrointestinal symptoms, suggesting a possible antecedent exposure rather than a confirmed active infection. The diagnosis of MFS with GBS overlap was retained. The patient was treated with intravenous immunoglobulin at a total dose of 2 g/kg, with close respiratory, bulbar, and autonomic monitoring and early rehabilitation. The outcome was favorable, with recovery of independent walking at one month and complete neurological recovery at six months. This case highlights the importance of considering MFS with GBS overlap in very young children presenting with acute gait disturbance and ocular motor signs.

## Introduction

Miller Fisher syndrome (MFS) is an uncommon variant of Guillain-Barré syndrome (GBS), classically defined by the triad of ophthalmoplegia, ataxia, and areflexia [1]. The discovery of anti-ganglioside GQ1b (anti-GQ1b) immunoglobulin G (IgG) antibodies has expanded this entity into the broader anti-GQ1b antibody syndrome spectrum, which includes MFS, Bickerstaff brainstem encephalitis, GBS with ophthalmoplegia, and overlap forms [2]. Pediatric MFS is rare, and diagnosis may be particularly challenging in very young children because gait disturbance, refusal to walk, and ocular misalignment may be the main presenting features [3]. MFS-GBS overlap should be considered when the classic features of MFS are associated with limb weakness and electrophysiological evidence of acute polyradiculoneuropathy [1,4]. Early recognition is important because patients may require immunotherapy and close monitoring for respiratory, bulbar, and autonomic complications [4].

We report a rare case of MFS with GBS overlap in a 20-month-old girl presenting with acute progressive gait disturbance, right abducens nerve palsy, generalized areflexia, motor-predominant polyradiculoneuropathy, and positive anti-GQ1b and anti-ganglioside GT1a (anti-GT1a) IgG antibodies.

## Case presentation

A 20-month-old girl, the youngest of three siblings, was admitted to our department for acute progressive gait disturbance over seven days associated with ocular deviation (Table 1). She had no significant personal medical history. There was no family history of neurological, neuromuscular, autoimmune, or metabolic disease. The pregnancy had been regularly followed and carried to term. Delivery was uneventful, with good neonatal adaptation. Her previous psychomotor development was considered normal for age, with the acquisition of expected motor milestones, including independent sitting and walking, before the current illness. Her immunization status was up to date according to the national vaccination schedule. There was no history of recent vaccination, unusual medication intake, toxic exposure, honey ingestion, or trauma.

**Table 1:** Clinical timeline of the patient's presentation, investigations, treatment, and follow-up CSF: cerebrospinal fluid; ENMG: electroneuromyography; IgG: immunoglobulin G; MRI: magnetic resonance imaging; anti-GQ1b: anti-ganglioside GQ1b; anti-GT1a: anti-ganglioside GT1a

Time point	Main events
Before illness	Normal pregnancy, term delivery, normal psychomotor development, and independent walking before illness
Day -7	Onset of progressive gait instability, repeated falls, and difficulty maintaining balance
Day -1	Inability to stand or walk without support, associated with convergent strabismus more marked on the right side
Admission	Severe axial ataxia, generalized areflexia, symmetrical limb weakness, and ophthalmologically confirmed right abducens nerve palsy
Hospitalization	CSF showed mild albuminocytologic dissociation; brain and spinal MRI were normal; ENMG showed acute motor-predominant polyradiculoneuropathy
Immunological work-up	Positive anti-GQ1b and anti-GT1a IgG antibodies; positive *Campylobacter jejuni* serology
Treatment	Intravenous immunoglobulin at 0.4 g/kg/day for five days, with close monitoring and early rehabilitation
Discharge	Improved sitting stability and assisted standing; walking still required support
One-month follow-up	Recovery of independent walking with marked improvement of ocular alignment
Six-month follow-up	Complete clinical recovery

The symptoms began seven days before admission with progressive gait instability. Her parents initially noticed unsteady walking, repeated falls, and difficulty maintaining balance. The condition progressively worsened, leading to refusal to walk and then inability to walk without support. One day before admission, she became unable to stand or walk. This motor deterioration was associated with the onset of convergent strabismus, more marked on the right side. She was first evaluated by a private pediatrician and was subsequently referred to our department. The clinical course occurred in an afebrile context. There were no seizures, altered consciousness, vomiting, meningeal signs, respiratory symptoms, or sphincter disturbances. No relevant infectious contact was reported within the family, and no recent gastrointestinal symptoms were reported.

**Figure 1:**
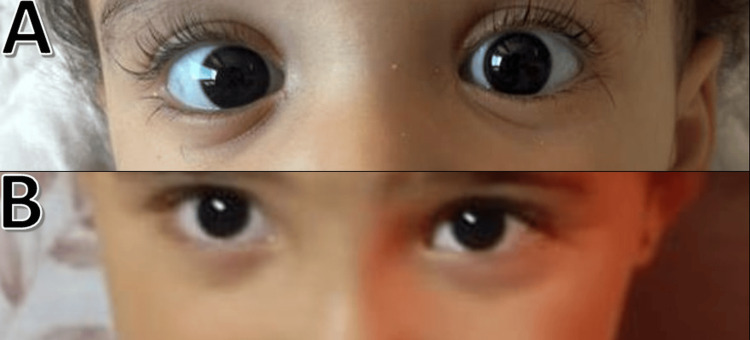
Ocular findings before and after treatment (A) Initial photograph showing right-predominant convergent strabismus, consistent with ocular motor involvement. (B) Photograph obtained at the six-month follow-up visit, showing complete resolution of ocular misalignment after treatment.

On admission, the child was conscious, alert, reactive, and afebrile, with a Glasgow Coma Scale score of 15/15. She was well-colored and well-hydrated, with no signs of respiratory distress. Vital signs were stable and appropriate for age. Oxygen saturation was maintained on room air. Her weight was 12 kg, and her height was 82 cm, consistent with normal growth for age. General physical examination showed no skin rash, peripheral lymphadenopathy, hepatosplenomegaly, or signs of dehydration. Cardiovascular, respiratory, and abdominal examinations were unremarkable. Neurological examination revealed an awake and interactive child with appropriate behavior for age. There was no irritability, drowsiness, behavioral change, or impairment of consciousness. No meningeal syndrome was observed. Postural assessment showed an inability to stand or walk independently. Sitting was impossible or markedly unstable without support, with significant axial hypotonia. When held in a sitting position, she showed marked trunk instability, consistent with axial ataxia. Formal muscle strength grading using the Medical Research Council scale could not be reliably performed because of the patient's young age and limited cooperation. True limb weakness was clinically suspected based on globally reduced spontaneous active movements in all four limbs, impaired limb motor activity beyond axial instability, generalized areflexia, and the absence of motor asymmetry. Severe axial ataxia clearly contributed to the inability to sit, stand, or walk without support, but the associated reduction in spontaneous limb movements suggested an additional symmetrical motor deficit. No abnormal movements, fasciculations, or fluctuating fatigability were observed. Deep tendon reflexes were absent in all four limbs. Plantar responses were indifferent bilaterally. There was no clonus, Babinski sign, or other pyramidal sign. Cranial nerve examination revealed convergent strabismus, predominantly on the right side (Figure 1A). Ophthalmological examination confirmed right abducens nerve palsy, with limitation of right eye abduction. Pupils were equal and reactive to light. There was no ptosis, facial palsy, swallowing disorder, dysphonia, hypersalivation, or tongue deviation. Coordination testing was difficult because of the patient's age. Nevertheless, the severe axial instability, inability to sit without support, and inability to stand were suggestive of severe ataxia. Gait could not be assessed at admission. Sensory examination was limited, but there was no obvious sensory level, asymmetrical response to painful stimulation, or apparent allodynia. There were no signs of bulbar involvement. The child had no choking episodes, dyspnea, ineffective cough, or bronchial congestion. There were no clinical signs of dysautonomia, including blood pressure instability, arrhythmia, profuse sweating, or urinary retention.

Given the acute gait disturbance, ocular motor involvement, generalized areflexia, and symmetrical limb weakness, the initial differential diagnosis included MFS, GBS, MFS with GBS overlap, Bickerstaff brainstem encephalitis, acute post-infectious cerebellitis, acute disseminated encephalomyelitis, acute myelitis, posterior fossa tumor or other structural lesion, infantile myasthenia, infant botulism, acute myopathy or myositis, and metabolic or toxic causes of acute paralysis. Initial laboratory investigations showed no major abnormalities (Table 2). Complete blood count, serum electrolytes, blood glucose, renal function, and liver function tests were within normal limits according to the available results. C-reactive protein was not significantly elevated. Creatine phosphokinase levels were normal. These findings argued against severe systemic infection, electrolyte disturbance, or primary muscle disease as the main cause of the acute neurological presentation.

**Table 2:** Initial laboratory investigations

Parameter	Result	Pediatric reference range	Interpretation
Hemoglobin	12 g/dL	10.5-13.5 g/dL, 6 months-2 years	Normal
Hematocrit	35%	33-39%, 6 months-2 years	Normal
Mean corpuscular volume	78 fL	70-86 fL, 6 months-2 years	Normal
White blood cell count	9.8×10^9^/L	5-15.5×10^9^/L, 1-2 years	No leukocytosis
Neutrophils	3.1×10^9^/L	1.27-7.18×10^9^/L, girls aged 6-23 months	Normal
Lymphocytes	5.4×10^9^/L	1.52-8.09×10^9^/L, girls aged 6-23 months	Normal for age
Platelets	350×10^9^/L	214-459×10^9^/L, girls aged 6-23 months	Normal
Sodium	139 mmol/L	135-145 mmol/L, ≥1 year	Normal
Potassium	4.4 mmol/L	3.6-5.2 mmol/L, ≥1 year	Normal
Chloride	105 mmol/L	102-112 mmol/L, 1-17 years	Normal
Bicarbonate	22 mmol/L	18-25 mmol/L, girls aged 1-3 years	Normal
Blood glucose	88 mg/dL	70-140 mg/dL, ≥1 year	Normoglycemia
Blood urea nitrogen	12 mg/dL	7-20 mg/dL, 1-17 years	Preserved renal function
Creatinine	0.32 mg/dL	0.19-0.49 mg/dL, girls aged 1-5 years	Preserved renal function
Total calcium	9.8 mg/dL	9.3-10.6 mg/dL, 1-17 years	Normal
Aspartate aminotransferase	32 U/L	8-50 U/L, girls aged 1-13 years	Normal
Alanine aminotransferase	18 U/L	7-45 U/L, girls ≥1 year	Normal
Alkaline phosphatase	230 U/L	142-335 U/L, 1 to <10 years	Normal for age
Total bilirubin	0.3 mg/dL	0-1.0 mg/dL, 15 days-17 years	Normal
Albumin	4.3 g/dL	3.5-5 g/dL, ≥12 months	Normal
Total protein	6.8 g/dL	6.3-7.9 g/dL, ≥1 year	Normal
C-reactive protein	1.8 mg/L	<5 mg/L, all ages	No significant inflammatory response
Creatine kinase	105 U/L	26-192 U/L, girls >3 months	Normal; no biological evidence of myositis or rhabdomyolysis

A lumbar puncture was performed during hospitalization approximately seven days after symptom onset (Table 3). Cerebrospinal fluid (CSF) was clear. Analysis showed 0 leukocytes/mm³ and a protein level of 0.53 g/L (reference: <0.45 g/L; age-adjusted pediatric reference interval from the local hospital laboratory). CSF glucose was preserved, and CSF culture was negative. These findings were consistent with mild albuminocytologic dissociation, supporting the diagnosis of an acute polyradiculoneuropathy within the GBS/MFS spectrum. Brain and spinal magnetic resonance imaging (MRI) were performed to exclude a central cause of the acute neurological syndrome, and neuroimaging was unremarkable, making posterior fossa tumor, structural brainstem encephalitis, acute disseminated encephalomyelitis, and acute myelitis less likely, with no evidence of cranial nerve enhancement, spinal nerve root thickening, or nerve root enhancement, without definitively excluding very early inflammatory central nervous system disorders.

**Table 3:** Cerebrospinal fluid, immunological, and infectious investigations SARS-CoV-2: severe acute respiratory syndrome coronavirus 2; IgG: immunoglobulin G; IgM: immunoglobulin M; anti-GQ1b: anti-ganglioside GQ1b; anti-GT1a: anti-ganglioside GT1a; anti-GM1: anti-ganglioside GM1; anti-GM3: anti-ganglioside GM3; anti-GD2: anti-ganglioside GD2; anti-GD3: anti-ganglioside GD3

Investigation	Result	Reference range/cut-off
Cerebrospinal fluid appearance	Clear	Clear
Cerebrospinal fluid leukocytes	0 cells/mm^3^	0-5 cells/mm^3^
Cerebrospinal fluid protein	0.53 g/L	<0.45 g/L
Cerebrospinal fluid culture	Negative	Negative
Anti-GQ1b IgG	Positive	-
Anti-GT1a IgG	Positive	-
Anti-GM1 IgG	Positive	-
Anti-GM3 IgG	Positive	-
Anti-GD2 IgG	Positive	-
Anti-GD3 IgG	Positive	-
*Campylobacter jejuni *IgG	294.99 U/mL	<30 U/mL
*Campylobacter jejuni *IgM	254.16 U/mL	<60 U/mL
SARS-CoV-2 IgG	10.73	Positive if >1

Electroneuromyography was performed during hospitalization (Table 4), approximately eight days after symptom onset, because of suspected GBS. The study was performed in all four limbs and showed markedly reduced motor compound muscle action potential amplitudes in the median, ulnar, and fibular nerves, abnormal F-wave responses, proximal latency abnormalities, and preserved sensory responses in all four limbs. The overall pattern was interpreted as an acute motor-predominant polyradiculoneuropathy, without evidence of primary muscle disease or neuromuscular junction disorder. Definite conduction block was not retained because formal electrodiagnostic criteria for conduction block were not applied.

**Table 4:** Electroneuromyography findings CMAP: compound muscle action potential

Electrophysiological parameter	Findings
Motor conduction, upper limbs	Severely reduced median and ulnar CMAP amplitudes; moderate prolongation of distal motor latencies; prolonged response duration; no proximal conduction block at the elbows
F-waves, upper limbs	Absent
Motor conduction, lower limbs	Markedly reduced fibular nerve CMAP amplitudes. The right posterior tibial motor study showed a distal CMAP amplitude of 2.77 mV with distal stimulation at the foot and a proximal CMAP amplitude of 2.01 mV with proximal stimulation at the popliteal fossa, associated with marked proximal latency prolongation. Formal electrodiagnostic criteria for conduction block were not applied; therefore, no definite conduction block was retained
F-waves, lower limbs	Prolonged latencies
Sensory conduction	Normal sensory nerve conduction in all four limbs
Overall conclusion	Findings strongly in favor of an acute motor-predominant polyradiculoneuropathy, without evidence of primary muscle disease or neuromuscular junction disorder

Immunological testing for anti-ganglioside antibodies was performed in serum using a qualitative immunodot assay, reported by the laboratory as an immunodot generic assay (Table 3). The results were interpreted categorically according to the reporting laboratory's criteria rather than as numerical titers or positivity thresholds. Signal intensity was reported only when specified by the laboratory, and no confirmatory testing with an alternative method was performed. The assay was positive for anti-GQ1b IgG, anti-GT1a IgG, anti-ganglioside GM1 (anti-GM1) IgG, anti-ganglioside GM3 (anti-GM3) IgG, anti-ganglioside GD2 (anti-GD2) IgG, and anti-ganglioside GD3 (anti-GD3) IgG. This finding strongly supported a diagnosis within the anti-GQ1b antibody syndrome spectrum, including MFS, acute ophthalmoplegia-associated forms, and overlap forms with GBS. *Campylobacter jejuni *serology was positive (IgG: 294.99 U/mL; reference: <30 U/mL; immunoglobulin M (IgM): 254.16 U/mL; reference: <60 U/mL), despite the absence of recent gastrointestinal symptoms. This finding suggested a possible post-infectious trigger in the context of acute immune-mediated neuropathy. Stool culture or polymerase chain reaction (PCR) for *Campylobacter jejuni *was not performed. Severe acute respiratory syndrome coronavirus 2 (SARS-CoV-2) serology was performed as part of the infectious work-up for possible antecedent immune triggers. Testing was performed using an enzyme-linked fluorescent assay (ELFA) VIDAS assay (bioMérieux, Marcy-l'Étoile, France) and showed isolated SARS-CoV-2 IgG positivity, with an IgG ratio of 10.73, while SARS-CoV-2 IgM was negative, with an IgM ratio of 0.13; positivity was defined by the reporting laboratory as a ratio >1. The patient had not received SARS-CoV-2 vaccination, and this result was interpreted as evidence of previous exposure rather than recent SARS-CoV-2 infection.

Based on the association of acute gait disturbance, severe axial ataxia, convergent strabismus predominantly on the right side with ophthalmologically confirmed right abducens nerve palsy, generalized areflexia, symmetrical limb weakness, mild albuminocytologic dissociation, electroneuromyographic evidence of acute motor-predominant polyradiculoneuropathy, positive IgG anti-GQ1b and anti-GT1a antibodies, and normal brain and spinal MRI, the diagnosis of MFS with GBS overlap was retained. The diagnosis of MFS was supported by the association of ataxia, generalized areflexia, and confirmed ocular motor involvement. However, the presence of symmetrical limb weakness and electrophysiological findings consistent with acute motor polyradiculoneuropathy favored an overlap form with GBS rather than isolated MFS.

The patient received intravenous immunoglobulin (IVIG) at a dose of 0.4 g/kg/day for five days. Treatment was well-tolerated, with no immediate allergic reaction, hemodynamic complication, or notable adverse event. Supportive management included close monitoring of respiratory, bulbar, and autonomic functions, with regular assessment of respiratory rate, oxygen saturation, heart rate, blood pressure, and swallowing. Early motor physiotherapy was initiated, including passive mobilization, prevention of contractures, age-adapted motor stimulation, and progressive rehabilitation of posture, sitting, standing, and walking. Adequate hydration and nutritional support were provided. Oxygen therapy, non-invasive ventilation, and intubation were not required.

The hospital course was favorable after IVIG therapy and motor rehabilitation. Progressive improvement in axial tone and spontaneous motor activity was observed after treatment initiation. The child gradually became able to maintain a sitting position with support and subsequently showed improved sitting stability. During the following days, approximately 10 days after the completion of the five-day IVIG course, upper limb motor activity improved, with better grasping and more active movements. Lower limb motor function also improved, allowing progressive assisted weight-bearing. The convergent strabismus partially regressed during hospitalization. Deep tendon reflexes remained decreased during the first 10 days after treatment. No respiratory, bulbar, or dysautonomic complications occurred during hospitalization. The patient had no choking episodes, bronchial congestion, oxygen desaturation, blood pressure instability, or cardiac rhythm disturbance. At discharge, 15 days after the completion of the five-day IVIG course, corresponding to an approximately 21-day hospital stay, her neurological status had improved compared with admission. Sitting was possible with improved stability, assisted standing was possible, and walking still required support. Outpatient motor physiotherapy was continued after discharge.

During follow-up, neurological recovery continued progressively. Gait improved with the reduction of instability, and muscle strength gradually recovered. No neurological relapse was reported during the available follow-up period. At one month, the child had recovered independent walking, with marked improvement of ocular alignment. At the six-month follow-up, she had achieved complete neurological recovery, with normal independent gait, resolution of the right abducens nerve palsy and convergent strabismus (Figure 1B), and no neurological relapse. Repeat electroneuromyography was not performed during follow-up because of the favorable and complete clinical recovery.

## Discussion

MFS and GBS are increasingly recognized as part of a continuous spectrum of acute immune-mediated neuropathies rather than completely separate entities. The present case fulfills the core clinical definition of MFS, namely, acute ophthalmoplegia, ataxia, and generalized areflexia [1]. However, the presence of symmetrical limb weakness, inability to stand or walk, and electrophysiological evidence of acute motor-predominant polyradiculoneuropathy support an overlap with GBS rather than isolated MFS [1,4]. This distinction is clinically important because isolated MFS usually has a favorable spontaneous course, whereas MFS-GBS overlap may be associated with the progression of limb weakness and potential respiratory, bulbar, or autonomic complications requiring close monitoring and early immunotherapy [4,5]. GBS is rare in childhood, with reported pediatric incidence varying according to geographic region and study design. In a population-based pediatric study from Hong Kong, the estimated annual incidence of pediatric GBS was 0.62 per 100,000 children, and MFS accounted for 25.4% of pediatric GBS cases [5]. More broadly, MFS represents only a small proportion of all GBS cases, approximately 1-7% in Western countries and up to 15-25% in some Asian populations [1]. Pediatric MFS remains uncommon, and available data are mostly based on retrospective series and case reports [3]. In the pediatric comparative study by Jang et al., the mean age at onset was 9.8±6.5 years, and overlapping MFS was identified in 36.4% of pediatric MFS patients [3]. Compared with these data, the age of our patient, 20 months, emphasizes the unusual early onset of this overlap phenotype and supports the need to consider anti-GQ1b antibody syndrome even in infants and toddlers presenting with acute gait disturbance and ocular motor involvement.

The diagnostic challenge in our patient was amplified by her very young age. In toddlers, classic symptoms may not be expressed verbally, formal strength testing is often unreliable, and ataxia may manifest as gait instability, repeated falls, refusal to walk, or inability to sit or stand without support [3]. In this context, the combination of acute loss of independent walking, severe axial instability, right abducens nerve palsy, and generalized areflexia was highly suggestive of a disorder within the MFS-GBS spectrum. However, the classification as MFS-GBS overlap was not based solely on gait refusal or axial instability. True symmetrical limb weakness was clinically suspected because spontaneous active movements were globally reduced in all four limbs, without motor asymmetry, and because limb motor activity appeared impaired beyond the severe axial ataxia. This interpretation was further supported by generalized areflexia and by electrophysiological evidence of acute motor-predominant polyradiculoneuropathy with preserved sensory responses. This case, therefore, emphasizes that acute strabismus or isolated ocular motor palsy associated with gait refusal in a very young child should prompt the consideration of anti-GQ1b antibody syndrome, even in the absence of a clear infectious prodrome. The clinical phenotype in this patient is particularly consistent with the anti-GQ1b antibody syndrome spectrum. The concept of anti-GQ1b antibody syndrome includes classic MFS, acute ophthalmoplegia, Bickerstaff brainstem encephalitis, GBS with ophthalmoplegia, and overlap forms [2]. Our patient had no impaired consciousness, pyramidal signs, seizures, or brainstem encephalitic features, making Bickerstaff brainstem encephalitis less likely. In contrast, the association of ophthalmoplegia, ataxia, areflexia, and limb weakness placed her between classic MFS and GBS. The recent European Academy of Neurology/Peripheral Nerve Society (EAN/PNS) guideline also supports anti-GQ1b antibody testing when MFS is suspected while emphasizing the role of CSF analysis and electrodiagnostic testing in patients with suspected GBS [6]. In this very young child, the diagnostic work-up supported an immune-mediated disorder within the MFS-GBS spectrum and made the main central nervous system mimics less likely. The combination of mild albuminocytologic dissociation without pleocytosis, unremarkable brain and spinal MRI, motor-predominant polyradiculoneuropathy with preserved sensory responses, and anti-ganglioside antibody positivity was concordant with the clinical phenotype. CSF findings were supportive but not independently diagnostic, as albuminocytologic dissociation may be absent or mild early in MFS and related anti-GQ1b syndromes [1,7]. Similarly, normal MRI findings reduced the likelihood of structural central nervous system lesions and inflammatory mimics, although they did not definitively exclude very early inflammatory disorders. Electrophysiology was particularly useful in supporting peripheral motor involvement in addition to severe ataxia. Given the early timing of the electrophysiological study and the absence of serial testing, the abnormalities were best interpreted as a motor-predominant polyradiculoneuropathy rather than a definitive electrophysiological subtype. Taken together, these findings reinforced the diagnosis of MFS-GBS overlap rather than isolated MFS when interpreted in the appropriate clinical context [6].

The antibody profile further strengthened the diagnosis. Anti-GQ1b IgG antibodies are strongly associated with ophthalmoplegia in MFS and related syndromes [1,2]. The presence of anti-GT1a IgG is also relevant because anti-GT1a may coexist with or cross-react with anti-GQ1b antibodies, and pediatric anti-GQ1b syndrome cohorts have reported anti-GT1a as one of the most frequent additional anti-ganglioside antibodies [7]. In this child, positivity for anti-GQ1b and anti-GT1a antibodies was concordant with the ocular motor involvement and the broader anti-GQ1b spectrum phenotype. Additional positivity for anti-GM1, anti-GM3, anti-GD2, and anti-GD3 antibodies suggests a broader anti-ganglioside immune response. However, because the test was qualitative, numerical positivity thresholds were not provided, signal intensity was only reported when specified by the laboratory, and no confirmatory testing with an alternative method was performed. Therefore, the broader antibody profile should be interpreted with caution. Because anti-ganglioside antibodies may cross-react through shared carbohydrate epitopes, each antibody should not be assigned a separate clinical manifestation without caution [7,8]. The anti-GM1 positivity may be compatible with the motor-predominant GBS component, but it does not by itself define the electrophysiological subtype. The positive *Campylobacter jejuni *serology provides a plausible post-infectious trigger. *Campylobacter jejuni *is one of the best-established infectious antecedents of GBS, particularly in cases associated with anti-ganglioside antibodies, through molecular mimicry between bacterial surface structures and peripheral nerve gangliosides [8,9]. The absence of recent diarrhea does not exclude this association, as antecedent infections may be clinically mild, forgotten, respiratory rather than gastrointestinal, or no longer symptomatic by the time neurological symptoms appear; however, positive serology alone does not establish active or recent *Campylobacter jejuni *infection, particularly in the absence of gastrointestinal symptoms and without confirmatory stool culture or PCR testing [8]. In our patient, positive IgM and IgG serology suggested recent or recent-past exposure, but stool culture or PCR was not performed. Therefore, *Campylobacter jejuni *should be presented as a probable or possible trigger rather than a proven cause. Similarly, isolated SARS-CoV-2 IgG positivity was considered of limited diagnostic relevance. The patient had not received the SARS-CoV-2 vaccination, and SARS-CoV-2 IgM was negative. In the absence of recent symptoms suggestive of SARS-CoV-2 infection, PCR confirmation, positive IgM, or a clear temporal relationship with the neurological presentation, this result was interpreted as evidence of previous exposure rather than recent infection, and no causal relationship with the neurological syndrome could be established.

Treatment with IVIG was justified by the overlap phenotype and loss of ambulation. Although isolated MFS is often self-limited, the presence of limb weakness and electrophysiological polyradiculoneuropathy places the patient closer to the GBS treatment framework. The EAN/PNS guideline recommends IVIG at 0.4 g/kg/day for five days in patients unable to walk unaided within the appropriate time window after onset of weakness, with plasma exchange as an alternative in selected patients [6]. In this case, the use of IVIG at 0.4 g/kg/day for five days was therefore consistent with current GBS management principles. The absence of respiratory, bulbar, or autonomic complications during hospitalization was reassuring, but did not eliminate the need for close monitoring, as deterioration in GBS can occur after presentation [4,6]. Supportive care and rehabilitation were also important components of management. In young children, prolonged immobility may rapidly lead to deconditioning, contractures, feeding difficulties, and developmental regression. Early physiotherapy, passive mobilization, prevention of contractures, and progressive age-adapted motor rehabilitation are therefore essential [6]. In our patient, improvement in axial tone, sitting stability, spontaneous limb movement, assisted standing, and eventually independent walking occurred progressively during follow-up while supportive care and structured rehabilitation were provided. The partial early regression of the convergent strabismus followed by complete resolution at six months is also consistent with the generally favorable ocular motor outcome described in MFS and anti-GQ1b antibody syndromes [1,3,7]. The prognosis in this case was favorable, with independent walking recovered at one month and complete neurological recovery at six months. This course is consistent with pediatric MFS studies, in which most children recover completely, often faster than adults [3]. In a pediatric anti-GQ1b antibody syndrome cohort and systematic review, most children improved after immunotherapy, and the majority achieved complete recovery within one year [7]. The persistence of decreased reflexes during the early recovery period is not unexpected, as areflexia may recover later than gait and ocular motor function in MFS [1]. The absence of relapse during available follow-up is also reassuring, although recurrent or relapsing anti-GQ1b antibody syndromes have been described; therefore, clinical follow-up remains advisable, particularly after severe or overlap presentations [1,2].

## Conclusions

MFS with GBS overlap is rare in very young children and may be difficult to recognize because gait disturbance, refusal to walk, and ocular misalignment can be the main presenting features. This case highlights an unusually early presentation of MFS-GBS overlap in a 20-month-old toddler and supports the consideration of the anti-GQ1b antibody syndrome spectrum in young children presenting with acute ataxia, ophthalmoplegia, areflexia, and suspected limb weakness. The diagnosis was supported by positive anti-GQ1b and anti-GT1a IgG antibodies, mild albuminocytologic dissociation, and electrophysiological evidence of motor-predominant polyradiculoneuropathy. Complete neurological recovery occurred at six months after IVIG therapy, close monitoring, and rehabilitation, consistent with the generally favorable outcome reported in MFS and anti-GQ1b antibody syndromes.
